# Spatial Control of Rabies on Heterogeneous Landscapes

**DOI:** 10.1371/journal.pone.0000027

**Published:** 2006-12-20

**Authors:** Colin A. Russell, Leslie A. Real, David L. Smith

**Affiliations:** 1 Department of Zoology, University of Cambridge Cambridge, United Kingdom; 2 Department of Biology, Emory University Atlanta, Georgia, United States of America; 3 Fogarty International Center, National Institutes of Health Bethesda, Maryland, United States of America; University of Liverpool, United Kingdom

## Abstract

Rabies control in terrestrial wildlife reservoirs relies heavily on an oral rabies vaccine (ORV). In addition to direct ORV delivery to protect wildlife in natural habitats, vaccine corridors have been constructed to control the spread; these corridors are often developed around natural barriers, such as rivers, to enhance the effectiveness of vaccine deployment. However, the question of how to optimally deploy ORV around a river (or other natural barrier) to best exploit the barrier for rabies control has not been addressed using mathematical models. Given an advancing epidemic wave, should the vaccine be distributed on both sides of barrier, behind the barrier, or in front of it? Here, we introduce a new mathematical model for the dynamics of raccoon rabies on a spatially heterogeneous landscape that is both simple and realistic. We demonstrate that the vaccine should always be deployed behind a barrier to minimize the recurrence of subsequent epidemics. Although the oral rabies vaccine is sufficient to induce herd immunity inside the vaccinated area, it simultaneously creates a demographic refuge. When that refuge is in front of a natural barrier, seasonal dispersal from the vaccine corridor into an endemic region sustains epidemic oscillations of raccoon rabies. When the vaccine barrier creates a refuge behind the river, the low permeability of the barrier to host movement limits dispersal of the host population from the protected populations into the rabies endemic area and limits subsequent rabies epidemics.

## Introduction

An emerging paradigm for the spread of infectious diseases is that of a structured metapopulation; host populations are sub-divided into interconnected sub-populations that are linked by migration and well-mixed with respect to disease transmission [1]–[4]. Migration rates among local populations typically vary substantially, and this can affect connectivity of the populations from the pathogen's perspective. Connectivity in the epidemiological sense is determined by the migration of infected or infectious hosts, which is affected by the average age of the infected or infectious population, the distance separating sub-populations, landscape features that inhibit or direct movement, seasonality in birth rates, juvenile transmission, or contact rates, and the behavior of the hosts or vectors. Epidemics on interconnected patches often generate wave-like patterns of spread, as is the case, for example, in human epidemics of measles[5], influenza[6], and dengue[7] and wildlife epidemics of rabies[8] and Ebola[9].

Population structure and heterogeneous connectivity can undermine or enhance efforts to control infectious diseases and invasive species. An important practical question is how disease control can be best deployed to take advantage of the variable connectivity of the network and the structure of heterogeneous landscapes. Here, we explore this question for the ongoing raccoon rabies epidemic in North America. Rabies tends to exhibit wave-like spread with well-organized wave fronts; variability in the velocity of the wave front is associated with the geographic proximity of rivers[10], forest cover[11], and mountains. Molecular evidence also suggests that rivers are an effective natural barrier to rabies dispersal[12].

Human rabies cases have been effectively limited in the USA, Canada, and Europe through the vaccination of domestic animals, but exposure to bats and suspicious wildlife can generate large economic costs from post-exposure prophylaxis even in these controlled nations[13]. Rabies in wildlife associated with Red fox in Europe and raccoons in the US and Canada have been controlled using ORV. The vaccines are usually distributed to take advantage of natural barriers that limit the dispersal of raccoons, such as along rivers to enhance the natural effect of a barrier, or along the mouths of mountain valleys or where lakes naturally constrict movement. The spatial pattern of vaccine delivery around barriers is contingent upon the availability of funds (D Slate, *pers comm*). When sufficient funds and ORV baits are available, vaccines are distributed on both sides of a river, but when budget is limited, the vaccines have been distributed on one or the other side of the river barrier. Is there an optimal policy for the spatial distribution of vaccine effort that maximizes the effectiveness of barriers in reducing the likelihood of disease emergence in affected areas?

Here we introduce a new mathematical model for the dynamics of rabies on heterogeneous landscapes (Methods), and we use it to investigate vaccine deployment strategies around rivers. Mathematical modelling of control strategies for rabies in wildlife has produced a substantial body of literature[14]–[18]. While previous works have considered whether culling, vaccination, or sterilization with re-release are the most effective strategies, little theoretical work has been done to evaluate how control strategies can be used most effectively to enhance the effects of natural barriers.

## Results

The model presented here is more detailed than many previous models for raccoon rabies with respect to the underlying population dynamics of raccoons, for example, we incorporate age-structure. At the same time, the model is relatively simple and transparent. Adult raccoons occupy a home range (i.e. a patch). Raccoon births and juvenile dispersal occur during brief periods; raccoons are born during the winter and disperse the following fall. Juvenile mortality is extremely high during juvenile dispersal, but the juvenile period ends once a juvenile settles on a new home range and becomes an adult. In this model, settling is a density-dependent process; settling is less likely in home ranges that are already close to their local carrying capacity. Thus, in the absence of rabies, the raccoon populations are regulated by high mortality during the juvenile dispersal phase and long juvenile dispersal periods near carrying capacity. In this spatial model, local carrying capacity and migration rates during juvenile dispersal are potentially heterogeneous.

Simultaneously, the model tracks the dynamics of rabies. Juveniles and adults in one of four states: uninfected, infected but not infectious, infected and infectious (i.e. in the furious phase), or recovered and immune. We assume that the latent period is long and variable, that the furious phase is short and mostly fatal, and that transmission is only local. In this model, furious raccoons move among home ranges, but other raccoons remain fixed within their home range, except for dispersing juveniles. Thus, rabies moves around during the furious phase in juveniles and adults, and in latent juveniles during the maturation phase. Vaccination moves susceptible individuals directly into the recovered and immune class.

We assume that population density is homogeneous, but that there is a river that inhibits dispersal. We have simulated vaccination along the river. We compare two different scenarios; vaccination in front of and behind the river, i.e. the same side and opposite side of the epidemic, respectively. For initial epidemic control (first 3 years) situating vaccination either on the front-side or the back-side of the barrier appears to have similar effects (data not shown), but the long term consequences of vaccine placement vary dramatically. By considering longer term effects of vaccine placement (first 10 years) we see a striking difference in the frequency of epidemic cycles based on the location of the vaccine corridor relative to the river.

**Figure 1:**
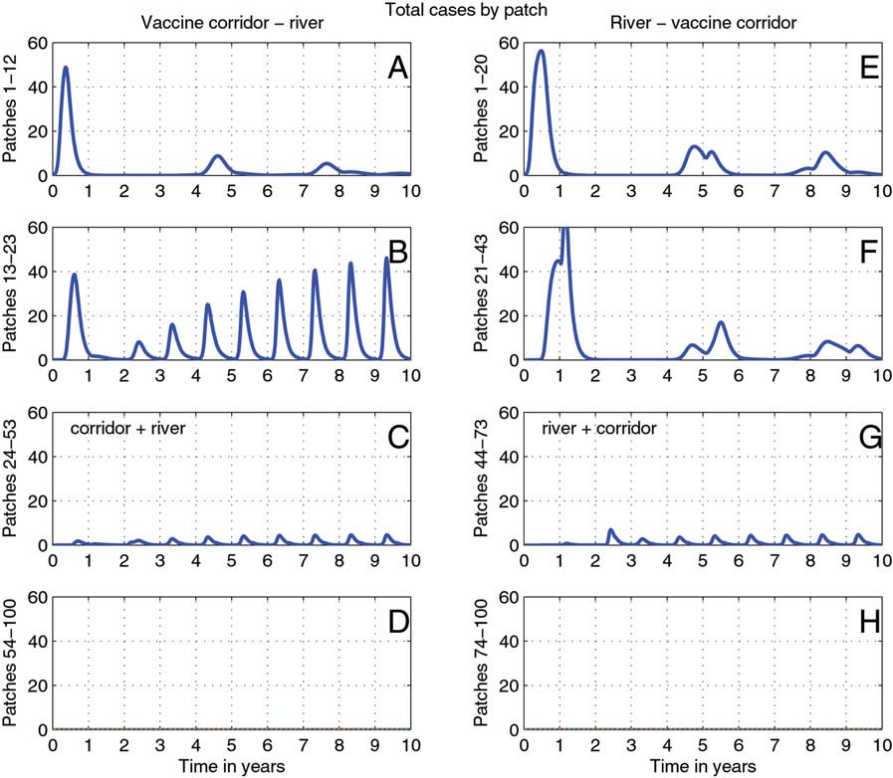
Time series for segments of the simulated landscape. (a–d) correspond to vaccines being placed in front of the river. (e–h) correspond to vaccines being placed after the river. (a) and (e) are the first patches to be initially infected in both scenarios and are the furthest from any impedances. (b) and (f) are the patches closest to the vaccine corridor and river, respectively. (c) and (g) comprise the patches which contain the vaccine corridor and river in each simulation. (d) and (h) are patches beyond the vaccine corridor and river.

**Figure 2:**
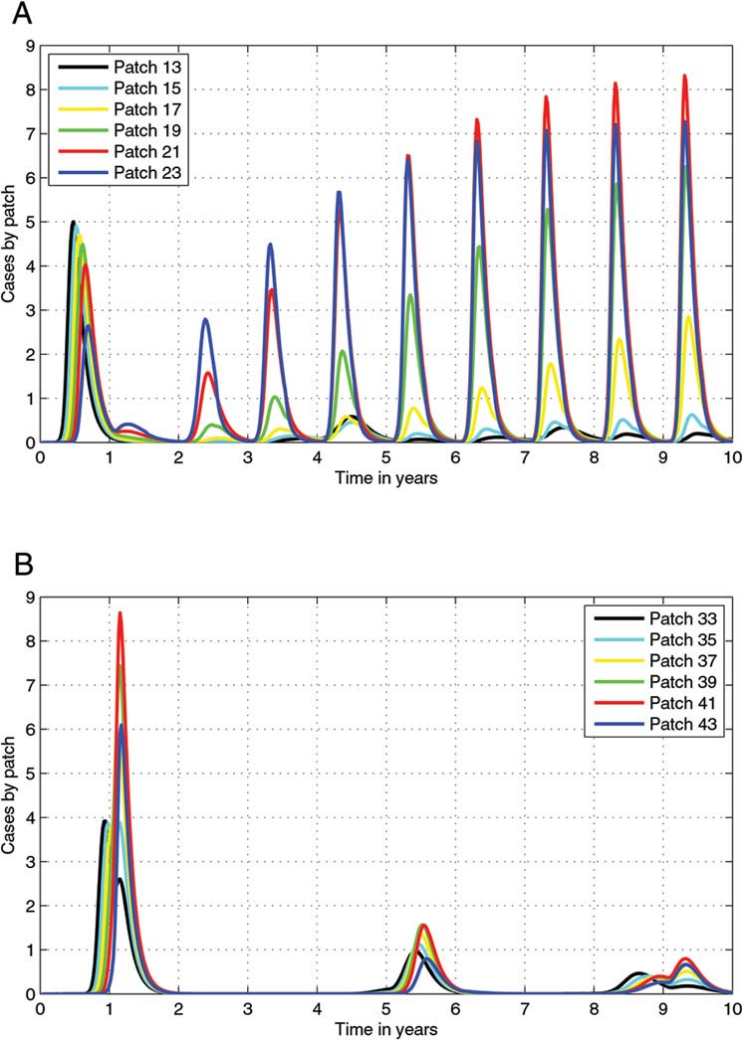
Time series for individual patches nearest (a) the vaccine corridor in the simulation with vaccines before the river and (b) the river in the simulation with vaccine corridor behind the river.

When vaccines were simulated behind the river, the epidemic front spread up to the river and exhausted local susceptible populations. Recruitment of large numbers of susceptibles from across the river was rare because the river prevented natural migration. As rabies is unlikely to drive the local population to extinction, surviving individuals reproduced and replenished susceptibles, so the disease circulates endemically at very low levels. Once the raccoon population reaches a critical density for rabies spread, smaller secondary waves of the disease return approximately every four years, as predicted by earlier models[19], [20]. Figure 1e,f shows the basic four year endemic cycles in the two sets of patches before the river and figure 1g shows low level disease cirulation within the *cordon sanitaire*. Examination of the disease incidence patterns of the individual patches nearest the river (figure 2b) shows little variation between indivdual patches.

**Figure 3:**
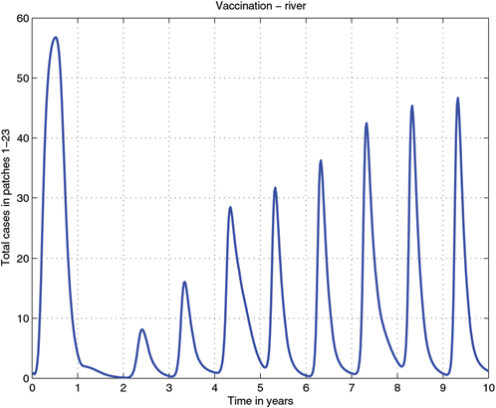
Time series for all patches before the vaccine corridor in the simulation with the vaccine corridor in front of the river.

However, when the vaccine corridor is situated in front of the river, we see annual cycles of increasing intensity (fig 1b, 2a, 3). While the host population outside the corridor is greatly reduced by rabies mortality, population density inside the corridor remains high. Juveniles migrate out of the corridor in search of less densely occupied home ranges each fall during the dispersal pulse. As vaccination levels inside the corridor are sufficient to induce herd immunity (but typically only 40–50% of the population become immunized [21], the migration of individuals (vaccinated and unvaccinated) from the corridor to the less densely populated unvaccinated areas is inevitable. Thus with every migration pulse the population density immediately outside the corridor increases sufficiently to generate new epidemics. As with the previous scenario, survivors from each epidemic will repopulate the rabies endemic area of the landscape until reaching a density sufficient for rabies spread. As no corridor is impenetrable, the increasing intensity of the epidemics immediately outside the corridor increases the risk of a breach during subsequent epidemics.

The placement of the vaccine corridor in front of the river primarily effects the demography of racccoons and rabies dynamics in patches immediately next to the river (fig 1a,b). Furthest from the vaccine corridor (fig 1a), the patches show a pattern of endemic disease cycling similar to the patches in the corridor, next to the river (fig 1e,f). In contrast, the patches closest to the vaccine corridor (fig 1b) show cycles of increasing intensity. Figure 2a further dissects this pattern and we see that the patches closest to the vaccine corridor also have the highest epidemic peaks which suggests that these epidemics are produced by the movement of susceptible (i.e. unvaccinated) individuals out of the vaccine corridor into patches where the disease remains endemic, or increased recruitment in those patches after being settled by dispersing but vaccinated juveniles.

**Figure 4:**
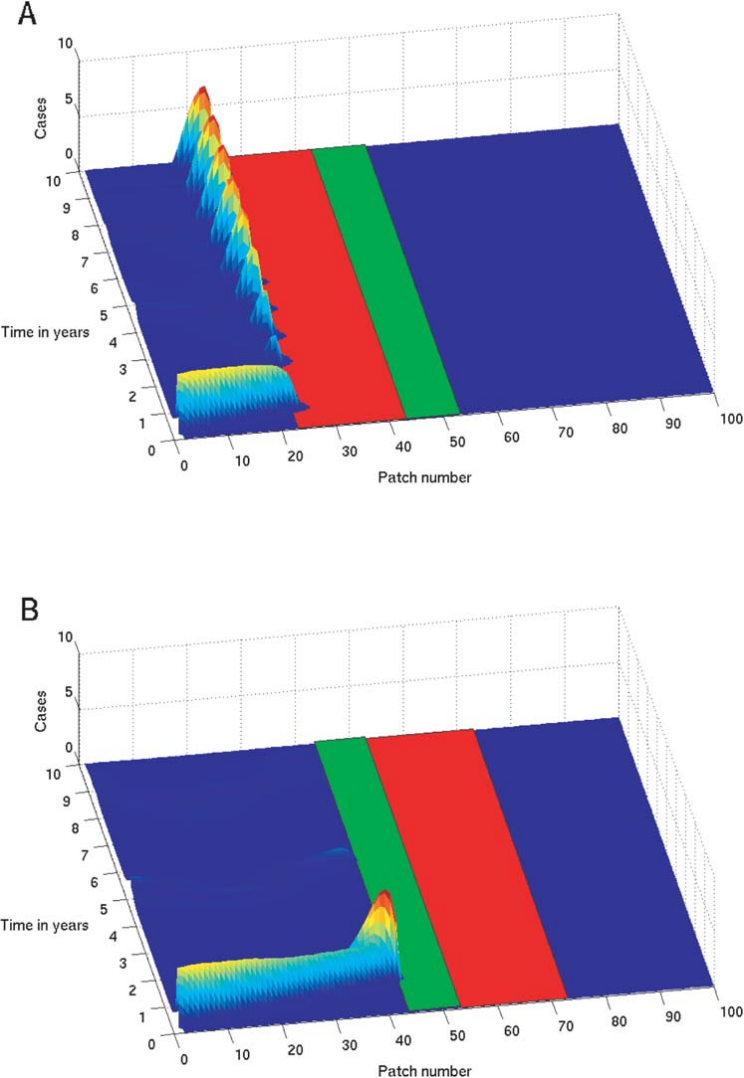
Disease incidence over space and time for simulations with vaccination before the river (a) and after the river (b). The river is represented by the light green bar and the vaccine corridor by the red bar.

We examined the overall pattern of disease incidence in the patches preceeding the vaccine corridor by summing the cases at each time step for each patch (figure 3). In addition to the annual epidemic peaks we also see a 4 year cycle in the depth of epidemic troughs. The decrease in trough depth corresponds to the build up of endemic cases over the landscape prior to 4th yearly endemic wave. Here the deepest troughs correspond to the combination of an endemic wave in the earliest patches and annual cycle immediately outside the vaccine corridor. The information in these figures is summarised by the projections in figure 4.

## Discussion

Our simulations suggest that even in an environment of sufficient funding, vaccinating on both sides of a natural barrier may not be the optimal strategy for rabies control. In the case of rabies, a disease that is usually fatal in its terrestrial carnivore hosts, the use of vaccines creates a demographic refuge. The densely populated refuge allows for the rapid recruitment of new individuals in nearby areas effectively sustaining the population birth rate and future epidemics. Thus, the demographic effects of vaccination and long term epidemiological consequences play into a vaccine strategy. Our results also apply to optimal control of other wildlife diseases that may strategically employ barriers, e.g. classic swine fever control in wild boar in Europe, or Ebola control in wild apes for both great ape conservation and public health. All else equal, our analysis suggests that the best strategy for reducing the likelihood of endemic disease cycles is to construct the vaccine corridor on the far side of any natural barrier.

We also wish to highlight the importance of the natural interplay between heterogeneity in connectivity and the epidemiology of wildlife diseases and diseases of humans, and the long term consequences of disease control strategies. In this case, a demographic refuge for the host also serves as a refuge for the pathogen, but the effects are not observable immediately. For example, the short term performance of the two simulated strategies are essentially indistinquishable for the first 3 years, but they differ substantially after 10 years. Subsequent endemic waves of the disease in the endemic areas present a long term hazard to fidelity of the *cordon sanitaire* as an increase in cases near the corridor can be loosely translated into a direct measure of the risk of breaching the *cordon sanitaire* and expansion of disease into previously uninfected geographic regions.

## Methods

Raccoon population biology in the northern Atlantic states is characterized by a birthing season during the winter and emergence of young in the spring, a period of growth during the summer when young animals stay with their mothers, and a period during the fall when juveniles disperse away from their natal territories[22], [23]. During the maturation phase, juveniles disperse until they establish a new home range. The seasonal birth pulse and the seasonal dispersal phase significantly affect both raccoon demography and the dynamics of control.

Here, we present a new, relatively simple and realistic mathematical model for raccoon rabies linking seasonality and host demography to rabies epidemiology and control. The model is an elaboration of previous mathematical models for rabies, but it includes the dominant features of the demography, the spring birth pulse and fall dispersal during maturation. We assume that, juveniles become adults when they establish a territory. Before territory establishment, juveniles continue to disperse and experience very high mortality rates. Maturation involves establishing a territory that is not already crowded, so the process of settling and maturing is density dependent, and regulates the population; juveniles continue to move until they find a patch that is not crowded–when the population density is near equilibrium, juveniles wander longer and experience higher mortality. This is the only regulatory mechanism we consider in this model, since we do not assume any density dependent mortality for adults, other than that induced by rabies.

For our model, we subdivide a continuous landscape into *i* patches arranged as one-dimensional linear array of cells (fig 5). Since we define the dispersal and movement structure across patches, the model could work just as appropriately for any connectivity of patches. In each cell we let *S_i_, J_i_, E_i_, L_i_, V_i_* and *R_i_*, denote the population density of adult and juvenile susceptible, adult and juvenile exposed, and adult and juvenile recovered/immune individuals. We let *I_i_* represent the density of infectious raccoons, including both juveniles and adults.

**Figure:**
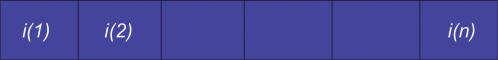
Landscape structure for model simulations. Movement is from neighbor to neighbor along a 1-dimensional linear array.

For heuristic purposes, we think of a raccoon as being in the *i*th patch if the patch contains the geographic center of the raccoon's home range. We assume that births occur at a constant per-capita rate *a(t)*, during the spring. Deaths occur at a constant per-capita rate *b* for adults and *j* for juveniles. During the maturation and dispersal period, described by the on-off function *M(t)*, juvenile mortality increases to *j+µ*. During this phase, juveniles leave a patch at the rate *φ*; they disperse from the *j*th patch to the *i*th patch at the rate *φ*κ*_i,j_*. Juveniles settle and mature at the density dependent rate , where *A_i_* is the density of adults, *A_i_ = S_i_+E_i_+R_i_*. This function makes settlement probability decline monotonically with adult density. In other words, the probability of settling and maturing in a patch is 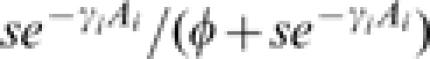, which declines with *γ_i_A_i_*.

We let 1/*σ* denote the average time from infection until a raccoon dies or recovers from rabies, *ρ* the fraction that become infectious, 1/α is the average time that a raccoon spends infectious, and ν the rate that raccoons are vaccinated. Infection occurs locally, at the rate βI_i_; infectious raccoons migrate out of their current patch at the rate ψ; they disperse from the *j*th patch to the *i*th patch at the rate ψκ*_i,j_*. Thus, except for juvenile dispersal during the maturation phase, infectious raccoons are the only ones that “move.”

The dynamics for juveniles are described by the following equations:
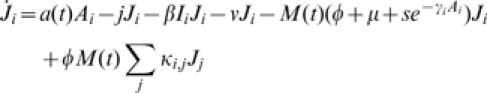


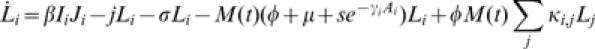


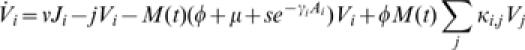



The dynamics for adults are described by the following equations:
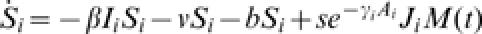


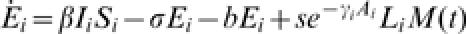


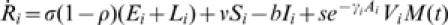



And finally, the dynamics of rabid individuals are described by a single equation:
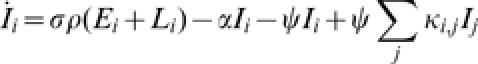



In addition to the assumptions made about raccoon population dynamics, we assume that any vaccine corridor constructed is effective, i.e. that it reduces the density of susceptible raccoons sufficiently to control the epidemic. In reality, corridors are somewhat porous, but they may remain effective for years, but they can be breached by a rare event, as in the case of the Ohio *cordon sanitaire* [24]. We assume that baits are distributed uniformly within a corridor, and at sufficient density such that when deployed in conjunction with a natural barrier the spread of the disease into new areas is limited.

In these simulations we consider a landsacpe 100 patches in length. Initial conditions of the simulation placed 1 infected individual into patch 1 at week 1, The total time period executed in each simulation was 10 years.

We introduce vaccination in association with rivers, where a river occupies 10 patches in the middle of our landscape. As estimated from previous studies[10], [25] in the absence of a control strategy the rate of spread across a river decreases the normal rate 7-fold. In this model with vaccination on either side of the river, spread is halted. The first three years of the epidemic show few differences between vaccinating before or after the river.
